# To Worry or Not: A Case of Nodular Pseudoangiomatous Stromal Hyperplasia of the Breast and a Review of the Literature

**DOI:** 10.7759/cureus.83678

**Published:** 2025-05-07

**Authors:** Aya Abdelrazzak, Wadih Ghaname, Pia Maria Obeid, Hanane Ziadeh

**Affiliations:** 1 Department of Obstetrics and Gynecology, Gilbert and Rose-Marie Chagoury School of Medicine, Lebanese American University, Beirut, LBN

**Keywords:** breast pathologies, fibroadenoma, nodular pash, pash, pseudoangiomatous stromal hyperplasia

## Abstract

Pseudoangiomatous stromal hyperplasia (PASH) is a benign mesenchymal proliferative breast lesion. It is very unusual for PASH to form a nodule on its own, in the absence of other breast lesions, an entity called “nodular PASH.” Given its rarity, we present an interesting case of this poorly understood breast pathology in a 25-year-old lady who was initially diagnosed with fibroadenoma of the left breast. Breast ultrasound and mammography are the most frequently used techniques to assess breast pathologies. Fibroadenomas can clinically and radiologically mimic the appearance of nodular PASH. Therefore, due to the non-specific features of nodular PASH, histologic examination remains the gold standard for confirming this rare diagnosis and ruling out low-grade angiosarcoma. Patients with nodular PASH have an excellent prognosis after excision. PASH is a benign stromal proliferation that histologically stimulates a vascular lesion; reporting additional cases is essential to develop comprehensive guidelines for its management.

## Introduction

Pseudoangiomatous stromal hyperplasia (PASH) is a benign mesenchymal proliferative lesion of the breast first described by Vuitch et al. in 1986 [1]. It is characterized by a complex network of slit-like spaces within the breast stroma that mimic vascular channels but are not lined by endothelial cells. PASH falls within the spectrum of benign stromal proliferations of the breast and is thought to arise from myofibroblasts.

Premenopausal women and menopausal women on hormone replacement therapy (HRT) are the most commonly affected by this entity [2]. Very few cases of PASH were found in males with gynecomastia [3]. The exact pathophysiology of PASH remains largely unknown, but given its prevalence mostly in premenopausal women, it is thought to be hormonally driven [4].

Clinically, PASH can manifest across a broad clinicopathologic spectrum, from an incidental histologic discovery to a clinically detectable mass [5]. Up to 23% of consecutive breast samples may contain incidental microscopic PASH [6]. However, it is quite uncommon for PASH to develop a nodule independently, without other lesions, a condition known as "nodular PASH" [7]. The prevalence of nodular PASH can be as low as 1-2% [8].

Fibroadenoma is considered the clinical and imaging mimicker of nodular PASH, which explains why many cases of nodular PASH can be mistaken for fibroadenoma of the breast [9]. Approximately 150 cases of PASH have been described in the literature. Therefore, given its rarity, we present an interesting case of this poorly understood breast pathology in a 25-year-old lady who was initially diagnosed with fibroadenoma of the left breast.

Surgery is usually recommended for patients with nodular PASH, especially if symptomatic or with a strong history of breast cancer. On the other hand, if PASH is histologically diagnosed, with no suspicious radiologic findings nor history of breast cancer, regular follow-ups and imaging can be considered, especially if the patient is asymptomatic. PASH has an excellent prognosis, with a risk of recurrence reaching 21%, especially if no complete excision was done.

## Case presentation

This is the case of a 25-year-old nulliparous woman who presented to our clinic with an enlarging mass in her left breast. Her past medical and surgical history was unremarkable, with no known drug or food allergies. She was not using hormonal contraception, had regular menstrual cycles, and reported no family history of breast disease.

**Figure 1:**
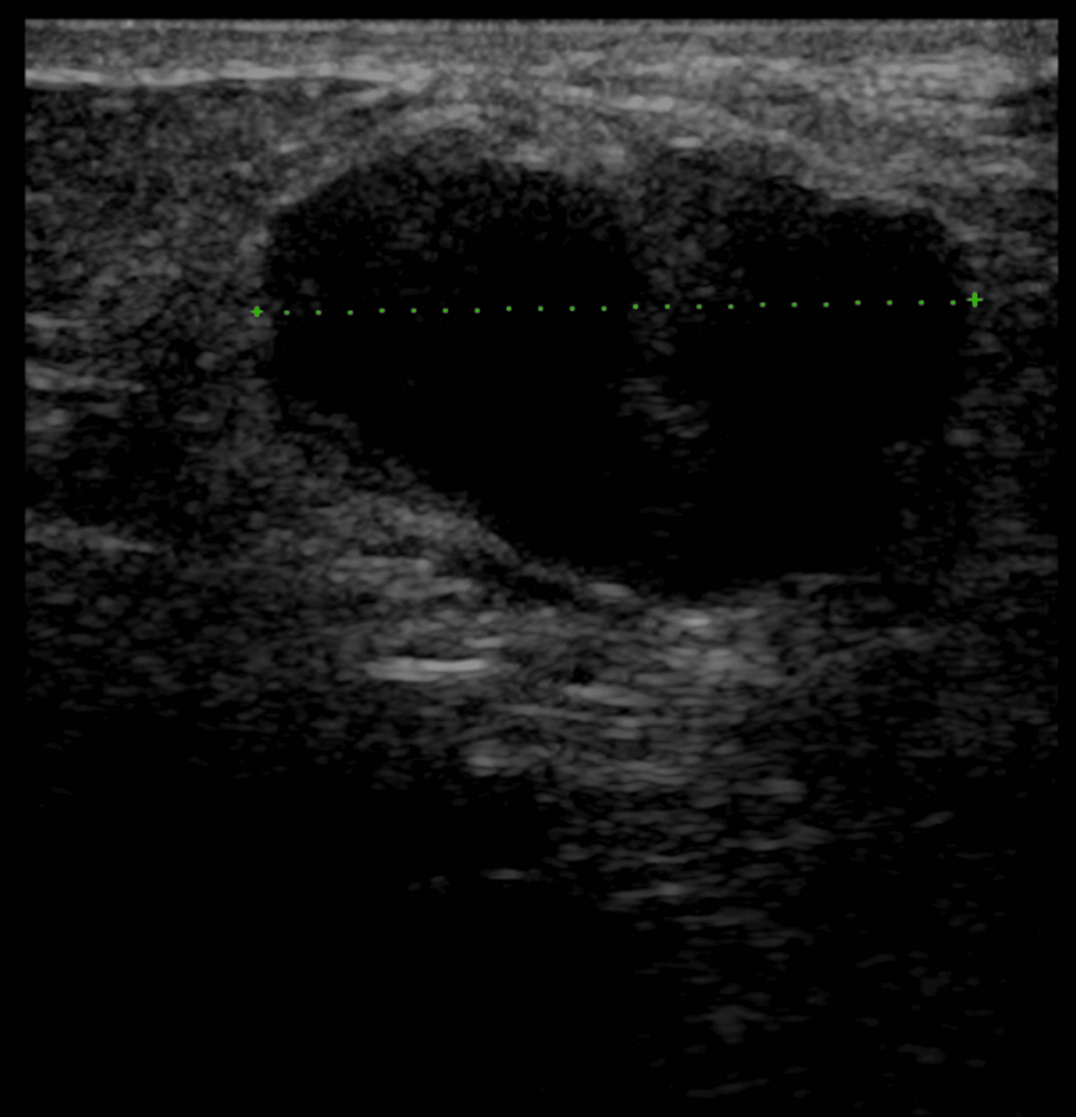
Hypoechoic lesion with lobulated contours measuring 2.7 cm in its longest axis at the level of the lower quadrant of the left breast primarily diagnosed as fibroadenoma (done in 2022)

The patient first visited our clinic in November 2022 after self-detecting a palpable lump in her left breast. Clinical examination revealed a firm, fixed 2 cm mass located at the 7 o’clock position of the left breast. No axillary or supraclavicular lymphadenopathy was noted. Breast ultrasound showed a hypoechoic lesion with lobulated contours, measuring 2.7 cm at its longest axis, in the lower inner quadrant at the 7 o’clock position, consistent with a fibroadenoma (Figure 1).

**Figure 2:**
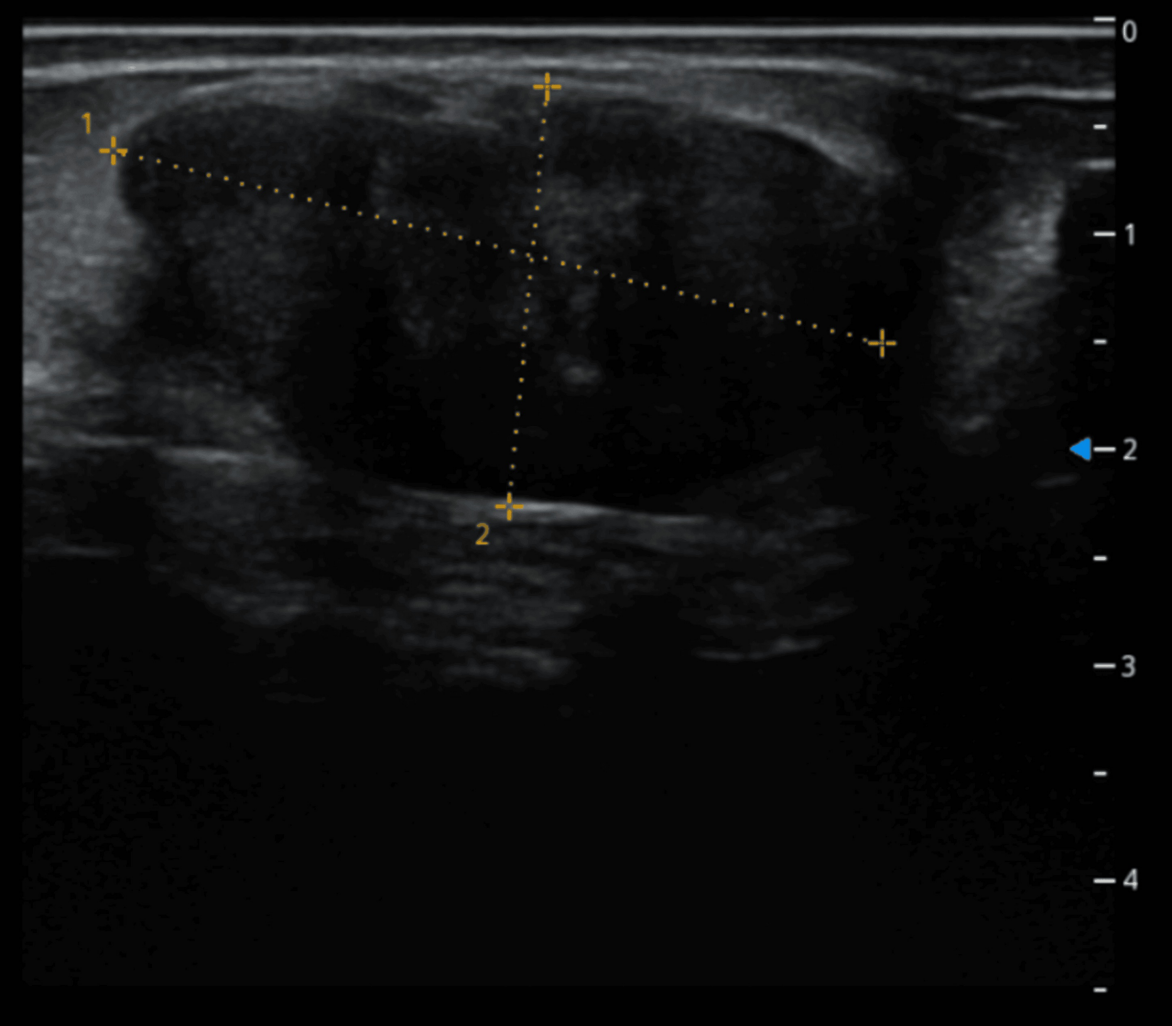
Homogeneous, well-defined, vascularized mass in the lower left quadrant of the breast measuring 36.7 x 19.5 mm (done in 2024)

Additionally, a 7 mm microcyst was identified in the internal para-areolar region. No axillary lymph nodes were detected, and the lesion was categorized as Breast Imaging Reporting and Data System (BI-RADS) 3. Based on these findings, a conservative follow-up approach was adopted with plans for periodic imaging every six to 12 months. However, the patient was lost to follow-up and returned to our clinic in March 2024, reporting an increase in the size of the previously detected lump. Repeat ultrasound revealed progression of the known lesion, now measuring 36.7 x 19.5 mm, with a homogeneous, well-defined, and vascularized appearance (Figure 2).

**Figure 3:**
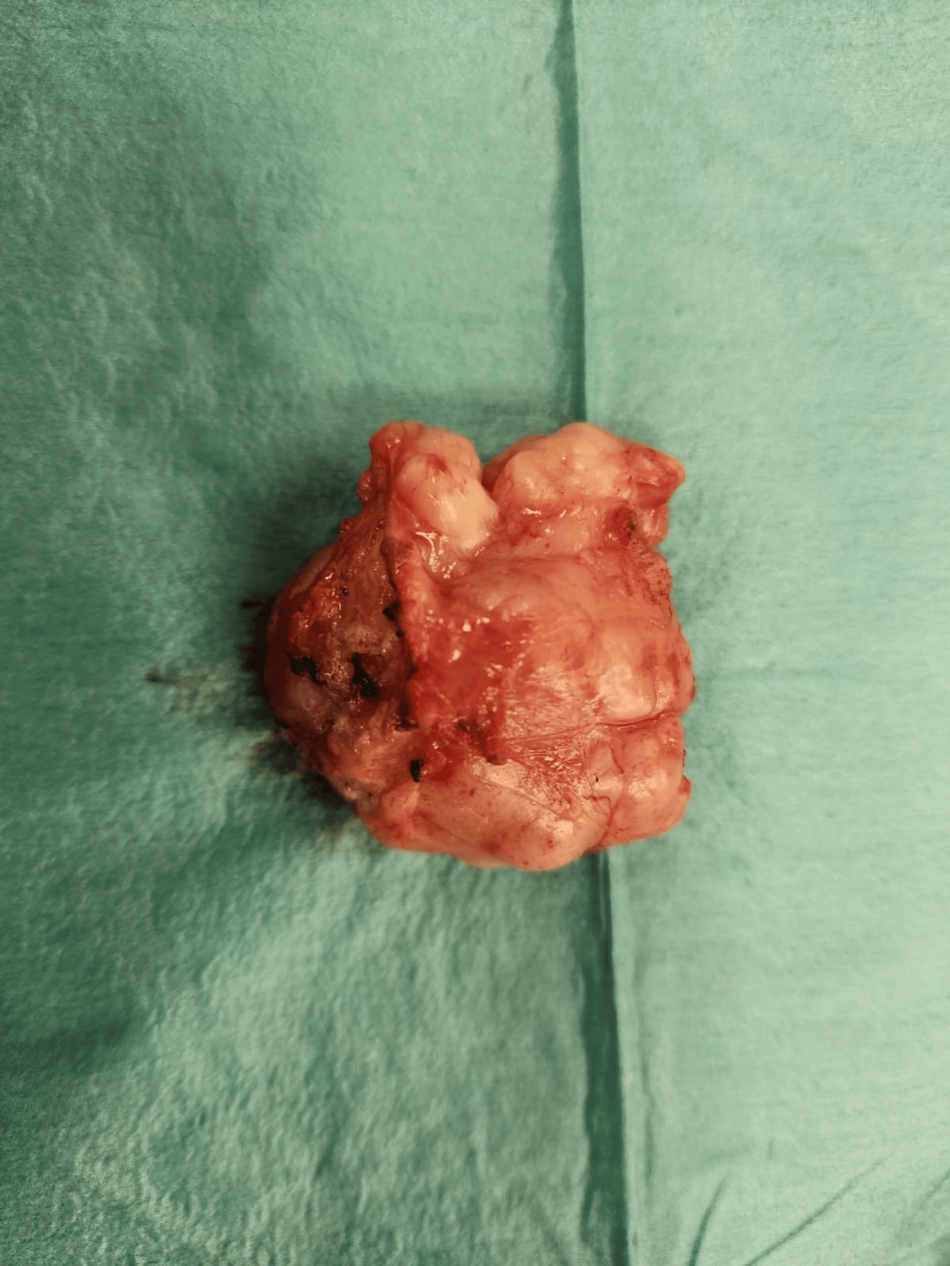
Firm, elastic breast tumor with multilobulated contours, corresponding to nodular PASH PASH: pseudoangiomatous stromal hyperplasia

Given the documented growth, a decision was made to surgically excise the lump, and histological analysis was planned following its removal. The lump was isolated from adjacent tissue during the surgery and resected under general anesthesia. The patient was discharged on day 1 postoperatively. The tumor (Figure 3) was sent to pathology, where they confirmed the diagnosis of nodular PASH, a rare benign stromal lesion of the breast. The diagnosis was based on the lesion's histologic features, which included dense stromal proliferation without cytologic atypia or invasive components.

## Discussion

PASH is a benign stromal proliferation that histologically simulates a vascular lesion [10]. It can have variable clinical presentations, often posing a diagnostic challenge. While it typically affects women in their 30s and 40s, it has also been reported in pediatric and postmenopausal patients, particularly those on HRT.

The lesion's growth patterns and its potential to mimic other breast pathologies highlight the importance of clinical awareness and appropriate management strategies [4]. Our case occurred in a 25-year-old lady who was initially diagnosed with fibroadenoma. Hence, clinical suspicion of PASH should be evaluated in conjunction with the patient's menopausal status and their use of HRT.

According to Kurt et al., most cases discovered had a tumor size of ≤2 cm [11]. In contrast, we reported a rare case that initially measured 2.7 cm and grew to 3.6 cm over two years.

The most common type of PASH is microscopic and usually asymptomatic. Some studies suggest that up to 23% of premenopausal women can have microscopic PASH on pathologic examination, often occurring concomitantly with other breast pathologies [6]. On the other hand, PASH as the primary pathologic finding or “nodular PASH” responsible for a solitary nodule is rare. This situation is exemplified by our patient, who was initially diagnosed with a fibroadenoma and later found to have an isolated nodular PASH.

Very few cases have documented PASH in males with gynecomastia [3]. Breast ultrasound and mammography are the most frequently used techniques to assess breast pathologies [12]. Given the age of our patient, breast ultrasound was chosen to evaluate her breast nodule.

The sonographic characteristics of PASH are generally nonspecific. Typically, PASH presents as a benign, hypoechoic, oval-shaped, well-defined mass [13]. These same features were seen in our case. However, given their nonspecific nature and overlap with other more common breast pathologies, our patient was initially diagnosed with fibroadenoma.

On mammography, PASH usually presents as a non-calcified, round, well-circumscribed mass or as a focal asymmetry [13]. Magnetic resonance imaging typically shows an isointense mass on T1-weighted gradient echo images. It may display a linear reticular "lacelike" pattern on axial T2-weighted images, suggesting the presence of slit-like spaces within the lesion [5].

Given the nonspecific radiologic features of PASH, histologic examination remains the gold standard for confirming this rare diagnosis and ruling out malignancy. Due to its relatively rapid growth over the past two years, we opted to directly excise the tumor without ordering additional imaging.

The pathologic findings of PASH typically consist of a round, rubbery, well-circumscribed mass, similar to the one we isolated in our case. Occasional cysts can be present [14]. On microscopy, PASH typically presents as a complex arrangement of slit-like spaces lined by endothelial-like spindle cells encased in dense collagenous stroma.

**Figure 4:**
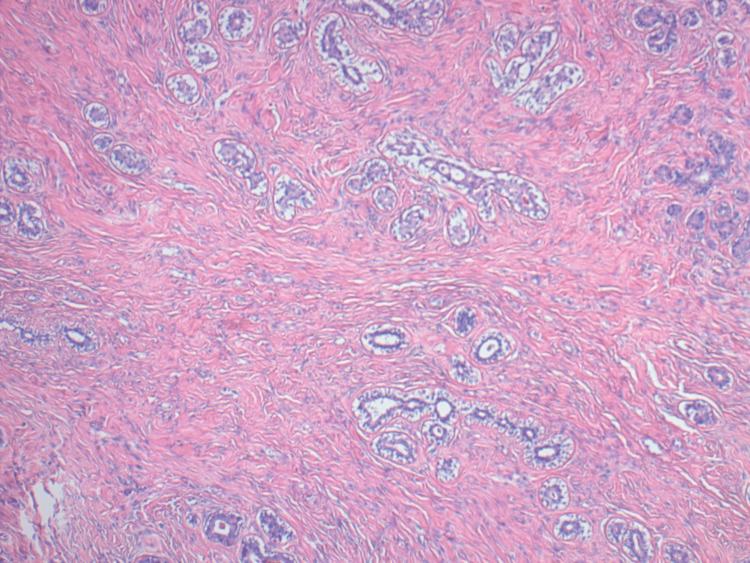
Section showing intermixed stromal and epithelial elements; the stroma exhibits anastomotic empty slit-like spaces within fibrous stroma with no evidence of atypia

Proliferation of fibroblasts and myofibroblasts, along with excessive collagen production, forms solid tissue interspersed with cystic areas that resemble dilated vessels [15]. These stromal cells are thought to have an excessive response to progesterone. These typical findings are consistent with the microscopic observations in our case (Figure 4).

Other histologic findings can be seen, as PASH presents a wide spectrum of histopathologic features. The differential diagnosis of PASH includes fibroadenoma, myofibroblastoma, mammary hamartoma, and angiosarcoma [16]. Angiosarcoma is the most critical differential diagnosis to exclude due to its malignant nature.

Grossly, angiosarcoma appears as an infiltrative, hemorrhagic mass with poorly defined borders. PASH and angiosarcoma exhibit anastomosing spaces lined by spindle cells on microscopic examination. However, differences become more apparent at different magnifications. On low-power microscopy, angiosarcoma disrupts the interlobular stroma and invades the surrounding fat, whereas PASH blends gradually with the adjacent stroma.

At high magnification, angiosarcoma spaces are lined by atypical endothelial cells showing mild to moderate pleomorphism, nuclear atypia, and increased mitotic activity. These cells vary in size and shape and have hyperchromatic nuclei. Hemorrhage with red blood cells may also be observed within these spaces. In contrast, the spindle cells in PASH are benign, lacking cytologic atypia or mitotic activity.

Immunohistochemical staining assists in distinguishing between the two conditions. Spindle cells in angiosarcoma are positive for endothelial markers such as CD31, CD34, and von Willebrand factor antigen. PASH spindle cells are positive for myofibroblastic markers such as CD34 and SMA but negative for endothelial markers [16].

In our case, the histologic features were classic for PASH, showing no evidence of cytologic atypia or invasive growth. A thorough evaluation and multidisciplinary discussion with our pathology team determined that immunostaining was unnecessary, as the diagnosis was evident based on morphology alone.

What makes this case particularly notable is its presentation as an isolated, enlarging nodular PASH in a young woman, with no concurrent breast pathology or significant risk factors. While PASH is typically an incidental and microscopic finding, this lesion presented as a distinct, palpable mass with progressive growth over two years, clinically and radiologically mimicking a fibroadenoma.

Ultrasound imaging revealed a well-defined, vascularized hypoechoic mass with lobulated margins, features highly suggestive of a fibroadenoma, illustrating the diagnostic challenge. Histologic examination confirmed the benign nature of the lesion, with no cytologic atypia, mitotic activity, or invasive features.

These findings underscore the importance of including nodular PASH in the differential diagnosis of any enlarging breast lesion in young women, even in the absence of hormonal therapy or a family history of breast cancer.

Regarding treatment, local excision is usually recommended for symptomatic patients with nodular PASH, those at high risk for breast cancer, enlarging lesions, and cases classified as BI-RADS 4 or 5 [17]. These lesions generally have an excellent prognosis, though recurrence rates range from 5% to 22%, particularly if the lesion is not completely excised [16]. This justifies the need for regular follow-ups.

Fortunately, PASH is not associated with an increased risk of malignancy [18]. However, it may coexist with malignant breast conditions such as ductal carcinoma in situ and invasive ductal carcinoma, which typically present with atypical radiologic findings [15].

For patients with a histologic diagnosis of PASH, no suspicious radiologic findings, and no history of breast cancer, regular imaging and follow-up can be considered, especially if the patient is asymptomatic [17].

Lastly, for patients experiencing enlarging or painful breast masses who decline surgery, medical management may be an option. Tamoxifen, a selective estrogen receptor modulator, has been reported to relieve symptoms in such cases. However, due to its anti-estrogenic side effects, tamoxifen use is generally limited to short durations [19].

## Conclusions

PASH is a benign stromal proliferation that histologically simulates a vascular lesion. It is a rare occurrence, especially of its nodal type. It usually occurs in premenopausal women or postmenopausal women on HRT and is thought to be hormonally driven. It can mimic several breast pathologies, and it is important to differentiate it from low-grade angiosarcoma. Immunohistochemistry could be helpful. Patients with nodular PASH have an excellent prognosis after excision. Finally, given the rarity of nodular PASH, reporting additional cases is essential to develop comprehensive guidelines for its management.
